# The Effect of Metformin on Ethanol- and Indomethacin-Induced Gastric Ulcers in Rats

**DOI:** 10.5152/tjg.2022.21195

**Published:** 2022-09-01

**Authors:** Betül Esra İpek, Meral Yüksel, Alev Cumbul, Feriha Ercan, Hülya Cabadak, Banu Aydın, İnci Alican

**Affiliations:** 1 Department of Physiology, Marmara University Faculty of Medicine, İstanbul, Turkey; 2 Marmara University Vocational Faculty of Health Services, İstanbul, Turkey; 3 Department of Histology and Embryology, Yeditepe University Faculty of Medicine, İstanbul, Turkey; 4 Department of Histology and Embryology, Marmara University Faculty of Medicine, İstanbul, Turkey; 5 Department of Biophysics, Marmara University Faculty of Medicine, Maltepe, İstanbul, Turkey

**Keywords:** Ethanol, gastric ulcer, indomethacin, oxidative stress, rat

## Abstract

**Background:**

Previous studies found metformin as an effective agent to suppress oxidative stress, inflammation, and apoptosis in various inflammatory diseases. The present study investigated the effect of metformin against 2 experimental gastric injury models in rats, using macroscopical, histopathological, biochemical, and immunostaining studies.

**Methods:**

**: **After 24 hours of fasting, male Sprague-Dawley rats (280-400 g) (n = 8 per group) received indomethacin (80 mg/kg; indo ulcer group) or absolute ethanol (5 mL/kg; ethanol ulcer group) or vehicle orally by gavage. Metformin (500 mg/kg) was given orally for 3 days prior to indomethacin or ethanol challenge. Ranitidine (50 mg/kg) was given orally for 3 days before indomethacin or ethanol administration as a positive control. On day 3, the animals were euthanized 6 hours after indo or 1 hour after ethanol challenge. Gastric samples were used for macroscopic scoring, histopathological examinations, and biochemical assays. Trunk blood was collected for the assessment of interleukin-1β level.

**Results:**

In both ethanol ulcer and indo ulcer groups, metformin decreased the extent of gastric lesions macroscopically and microscopically, improved the high chemiluminescence levels, and the percentage of terminal deoxynucleotidyl transferase dUTP nick end labeling (TUNEL)-positive apoptotic cells compared with untreated ulcer groups. Gastric blood flow analysis revealed significant increases in both metformin-treated ulcer groups compared to untreated ulcer groups.

**Conclusion:**

The findings of the present work demonstrated the gastroprotective effect of metformin against the development of gastric mucosal lesions induced by ethanol and indomethacin in non-diabetic, normoglycemic rats via its antioxidant and anti-apoptotic properties and partly from its ability to restore blood flow.

## Main Points

- Metformin has anti-inflammatory properties beyond its effect to control glycemia.
- Maintenance of redox balance is one of the key mechanisms of metformin as an anti-inflammatory agent.
- Metformin is gastroprotective against the development of gastric mucosal lesions induced by ethanol and indomethacin in non-diabetic, normoglycemic rats.

## Introduction

Metformin is a member of biguanides that improve insulin sensitivity in cases with type II diabetes mellitus. Previous studies found metformin as an effective agent to suppress oxidative stress, inflammation, and apoptosis in various inflammatory diseases.^1,2^ In a recent study, Samman et al.^3^ examined metformin’s effect on the severity of chronic colitis in rats challenged with dextran sodium sulfate and observed significant improvement in colonic lesions and oxidative stress markers. A similar beneficial effect was also reported in a rat model of acetic acid-induced colitis.^4^ The underlying mechanisms of metformin’s beneficial effects on various inflammatory settings include the suppression of reactive oxygen species (ROS) production, the release of pro-inflammatory mediators, expression of enzymes involved in the inflammatory cascade, and apoptosis.^5,6^

Stress, smoking, high alcohol consumption, chronic use of non-steroidal anti-inflammatory drugs (NSAIDs), and *Helicobacter pylori* infection are among the common exogenous agents that lead to gastric lesions via disrupting epithelial integrity. Non-steroidal anti-inflammatory drugs that are widely used for the treatment of rheumatoid arthritis and osteoarthritis may cause gastrointestinal injury primarily by inhibiting prostaglandin synthesis, inducing apoptosis, increasing inflammatory cell infiltration, and generating ROS.^7,8^

It is well known that ethanol ingestion causes severe tissue destruction which is seen as erosive hemorrhagic lesions, congestion in vessels, edema and necrosis in gastric mucosa. Disruption of the mucus barrier in gastric mucosa and cell damage due to oxidative stress, ROS formation, and increase in permeability are among the mechanisms of the action of ethanol as a necrotizing agent.^9^

Based on the above knowledge, the current work examined the effect of metformin on the development of gastric damage in two experimental models in rats, using macroscopical, histopathological, biochemical, and immunostaining studies.

## Materıal and Methods

### Animals

Male Sprague-Dawley rats (280-400 g) were housed in plexiglass cages under standard condition of temperature (20°C-26°C) and humidity (40%-60%). The animals were fed with standard pellet and water. The experimental procedures undertaken in this study were performed according to the criteria outlined by the Guide to the Care and Use of Laboratory Animals (National Institutes of Health Publications No. 8023, revised 1978). The study protocol was approved by Marmara University School of Medicine, Animal Care and Use Committee (no: 19.2019.mar; March 04, 2019).

Metformin and ranitidine were supplied from Cayman Chemical (Ann Arbor, Mich, USA). Indomethacin was purchased from Sigma Chemical Co. (St. Louis, Mo, USA). Ethanol was purchased from Merck (Darmstadt, Germany).

### Indomethacin-Induced Gastric Ulcers

After 24 hours of fasting, the rats (n = 8 per group) received indomethacin (80 mg/kg) (indo ulcer group) or the vehicle (5% sodium bicarbonate) (indo control group) orally.^10^ This high dose was chosen to produce severe gastric lesions, aiming to more clearly demonstrate the metformin’s potential effect. Metformin (500 mg/kg; peroral) was given for 3 days prior to indo challenge (MET-indo ulcer group). Ranitidine (50 mg/kg; peroral) was given for 3 days before indo administration to another group as a positive control (RTN-indo ulcer group).^11^ On day 3, 60 minutes after the last dose of metformin or ranitidine, the animals were administered with indomethacin and euthanized by cervical dislocation 6 hours after ulcer induction, and trunk blood was collected.

### Ethanol-Induced Gastric Ulcers

After 24 hours of fasting, the rats (n = 8 per group) received absolute ethanol (5 mL/kg) (ethanol ulcer group) or saline (ethanol control group) orally by gavage.^12^ Metformin (500 mg/kg; peroral) was given for 3 days prior to ethanol challenge (MET-ethanol ulcer group). Ranitidine (50 mg/kg; peroral) was given for 3 days before ethanol administration to another group as a positive control (RTN-ethanol ulcer group).^11^ On day 3, 60 minutes after the last dose of metformin or ranitidine, the animals were administered with ethanol and euthanized by decapitation 1 hour after ulcer induction and trunk blood was collected.

The dose of metformin (500 mg/kg) was chosen according to our preliminary study data where 3 doses of the drug (100 mg/kg, 200 mg/kg, and 500 mg/kg) were used. Our preliminary experiment also demonstrated that 500 mg/kg dose did not alter fasting blood glucose levels of rats in control and ulcer groups (data not shown).

In experiments with two gastric ulcer models, the stomach samples were used for macroscopic scoring, histopathological examinations, and biochemical assays. Trunk blood was collected, and the serums were used for the assessment of interleukin (IL)-1β level.

### Macroscopic Evaluation of the Gastric Damage

Stomach was incised along the greater curvature and rinsed with saline to remove stomach contents. The lengths of the lesions were measured in each stomach, and the sum of lengths (mm) was expressed as the “lesion length.” The stomach was also scored according to a previously described scale and expressed as the “lesion score”: 0 = no damage, 1 = blood in lumen, 2 = pinpoint erosions, 3 = 1-5 small erosions (<2 mm), 4 = >5 small erosions, 5 = large erosions (>2 mm), 6 = >3 large erosions.^13^

### Microscopic Evaluation of the Gastric Damage

Samples from the fundic region of the stomach were placed in 10% formaldehyde, dehydrated in ascending alcohol series (70%, 90%, 96%, and 100%), and embedded in paraffin. For each animal, 4 randomly chosen sections (5 µm) were stained with hematoxylin and eosin (H&E) and examined under an Olympus BX51 photomicroscope.

The gastric damage was examined using the following criteria: epithelial desquamation; mucosal congestion, focal necrosis, and hemorrhage; glandular damage; and inflammatory cell infiltration. Each criterion was scored using a scale ranging from 0 to 3 (0 = none, 1 = mild, 2 = moderate, and 3 = severe). The total score was 12.^14^

All microscopic examinations were performed by an experienced histologist who was unaware of the treatment groups (F.E.).

### Measurement of Gastric Glutathione Content

Gastric samples were homogenized in 10 volume of 10% trichloracetic acid and centrifuged at 3000 rpm for 15 minutes at 4°C. The supernatant was removed and recentrifuged at 10 000 rpm at 4°C for 8 minutes. Glutathione was determined spectrophotometrically using modified Ellman method.^15^

### Measurement of Gastric Superoxide Dismutase and Catalase Activities

Measurement of gastric superoxide dismutase (SOD) activity was performed in 50 mM potassium phosphate (pH = 7.8), 0.1 mM Ethylene Diamine Tetra Acetic Acid (EDTA), 0.2 mM riboflavin in 10 mM potassium phosphate (pH = 7.5), 6 mM *o*-dianisidin, and tissue extract containing cuvettes which were illuminated with 20-W Slylvania Grow Lux fluorescent tubes to maintain a temperature of 37°C. Superoxide dismutase activity was measured spectrophometrically at 460 nm and the results were expressed as U/mL.^16^

For the measurement of catalase activity, the absorbance for the mixture of 0.4 mL tissue homogenate and 0.2 mL H_2_O_2_ was recorded against the blank at 240 nm. The results were expressed as U/mL.^17^

### Measuring Reactive Oxygen Species Using Chemiluminescence Assay

Luminol and lucigenin probes were used for this assay. Luminol detects hydrogen peroxide, hydroxyl radical, hypochlorite, peroxynitrite, and lipid peroxyl radicals. Lucigenin detects superoxide radicals. Tissue chemiluminescence levels were recorded using Mini Lumat LB 9509 luminometer (EG&G Berthold, Germany). The counts were obtained at 1-minute intervals for 5 minutes. The results were expressed as area under the curve of relative light unit (rlu) for 5 minutes per mg tissue.^18^

### Evaluation of Apoptosis Using Immunohistochemistry

Gastric samples were immersed in 10% neutral formaldehyde in 0.1 M phosphate-buffered solution (pH = 7.4). Paraffin-embedded sections of 5 µm thickness were stained using a modified TUNEL technique.^19^ Each section was fractionated optically using the Stereo Investigator version 11.0 image analysis software (Microbrightfield, Colchester, Vt, USA). In each frame, a counting area was designated unbiasly, and the apoptotic index (%) was calculated as apoptotic cells/total cells × 100.

### Gastric Blood Flow

To perform the gastric blood flow experiments, another group of rats (n = 5 per group) was used. These experiments were performed in 4 groups: ethanol ulcer, indo ulcer, MET-ethanol ulcer, and MET-indo ulcer groups. Prior to decapitation, the rats were anesthetized with ketamine (100 mg/kg) and xylazine (10 mg/kg) intraperitoneally. After exposing the stomach by a midline incision of the abdomen, the gastric blood flow was measured using a laser-Doppler flowmeter (PeriFlux System 5000, Perimed, Sweden). A miniature probe was placed on the serosal surface of the corpus. After stabilization for 5 minutes, the mean value of the recording between 5 and 10 minutes was taken for the analysis and expressed as PU (perfusion unit).

### Measurement of Serum IL-1β Levels

Blood samples were centrifuged at 3000 rpm for 15 minutes. The supernatant was used for the measurement of IL-1β levels using rat immunoassay enzyme-linked immunosorbent assay kits (Thermo Fisher Scientific, Waltham, Mass, USA).

### Statistical Analysis

Data are expressed as mean ± SEM. All data were analyzed for normal distribution using the Shapiro–Wilk test. The histological data were compared using Mann–Whitney *U* non-parametric test. Other parameters were compared using two-way analysis of variance. Post hoc testing was completed using Tukey–Kramer multiple comparison tests, with significance set at *P* < .05. Calculations were done using Instat statistical analysis package (GraphPad Software, San Diego, Calif, USA).

## Results

### Severity of Gastric Damage at Macroscopic Level

**Figure 1.**
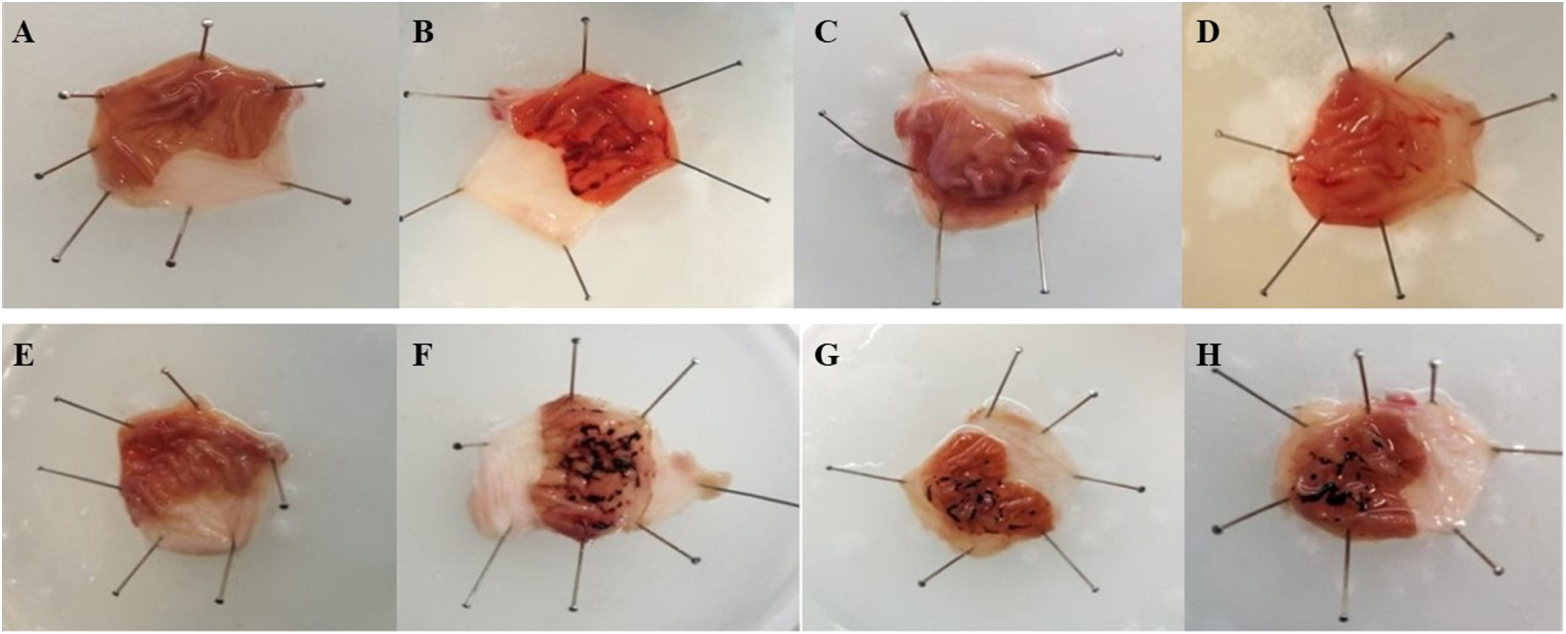
Representative photographs illustrating the gross macroscopic appearances of gastric samples from the experimental groups. Ethanol control (A), ethanol ulcer (B), metformin-treated ethanol ulcer (C), ranitidine-treated ethanol ulcer (D), indomethacin control (E), indomethacin ulcer (F), metformin-treated indomethacin ulcer (G), and ranitidine-treated indomethacin ulcer (H) groups.

In ethanol ulcer group, pretreatment with metformin caused a decrease in both lesion score (1.37 ± 0.90 vs 5.99 ± 0.01; *P* < .001) and lesion length (2.38 ± 1.69 mm vs 54.88 ± 8.11 mm; *P* < .001) in comparison to untreated ulcer groups. A similar result was observed in metformin-treated indo ulcer group. Metformin was effective to decrease the extent of lesions in this group in terms of lesion length (38.63 ± 10.56 mm vs 69.31 ± 6.67 mm; *P* < .05). Treatment with ranitidine decreased the severity of lesions in both ulcer models (Table 1 and Figure 1).

### Severity of Gastric Damage at Microscopic Level

**Figure 2.**
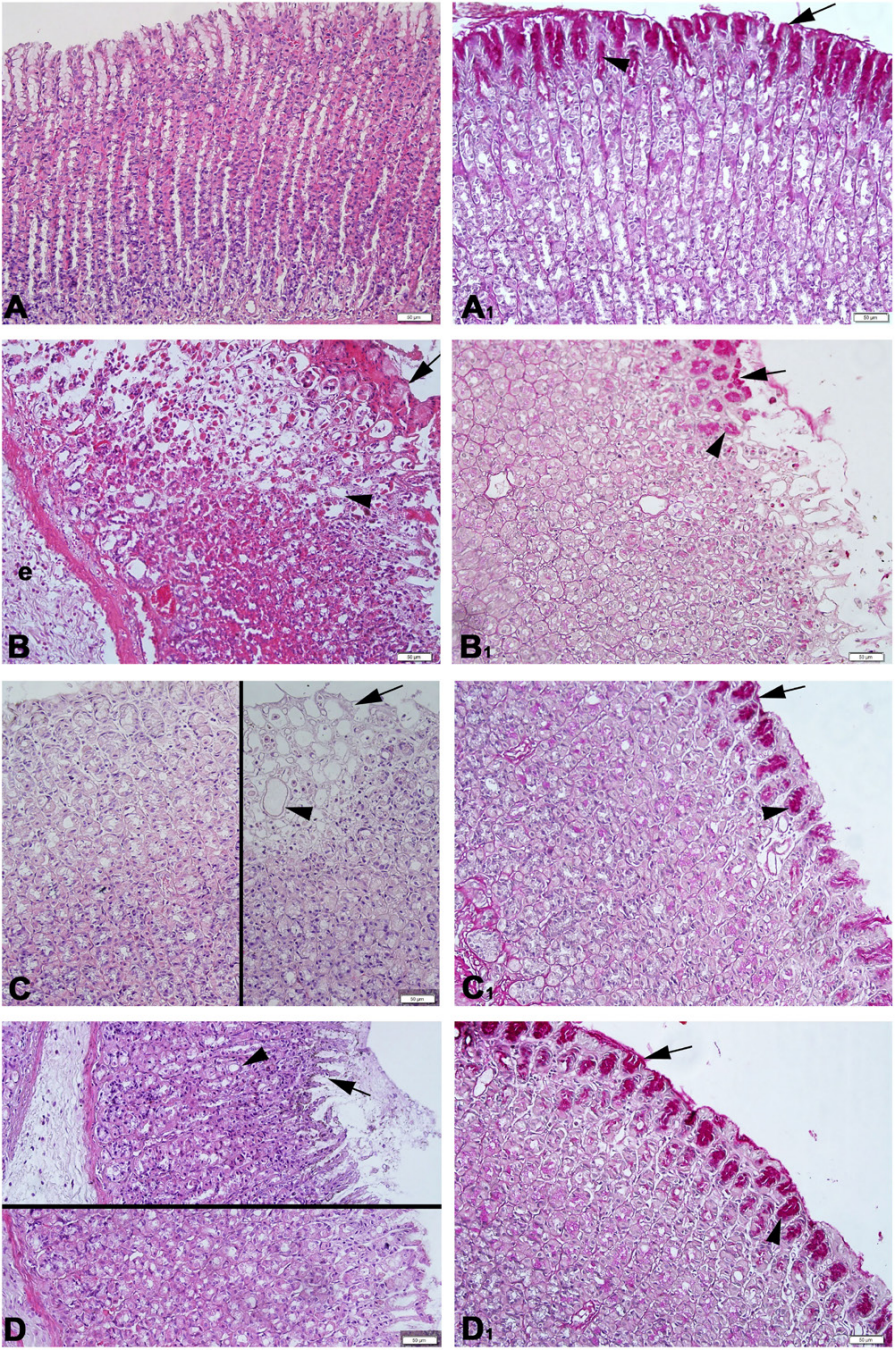
Representative micrographs illustrating the histological appearances of gastric samples from the ethanol groups. Micrographs A, B, C, and D depict H&E staining and A1, B1, C1, D1 depict PAS staining. Ethanol control group (A, A1) revealed a regular gastric surface epithelium and glandular cells with PAS-positive surface mucous cells (arrow) and neck mucous cells (arrowhead). In ethanol ulcer group (B, B1), there was severe degeneration in both surface cells (arrow) and glandular epithelial cells (arrow) with inflammatory cell infiltration (e). PAS-positive surface mucous cells (arrow) and neck mucous cells (arrowhead) were markedly decreased. RTN-ethanol ulcer group (C, C1) revealed surface epithelium (arrow) and glandular epithelium (arrowhead) with moderate degeneration and a moderate increase in PAS-positive surface mucous (arrow) and neck mucous cells (arrowhead). MET-ethanol ulcer group (D, D1) was comparable to RTN-ethanol ulcer group in terms of morphology but there was markedly increased PAS-positive surface mucous cells (arrow) and neck mucous cells (arrowhead). Scale: 50 µm. Original magnification 10×.

**Figure 3.**
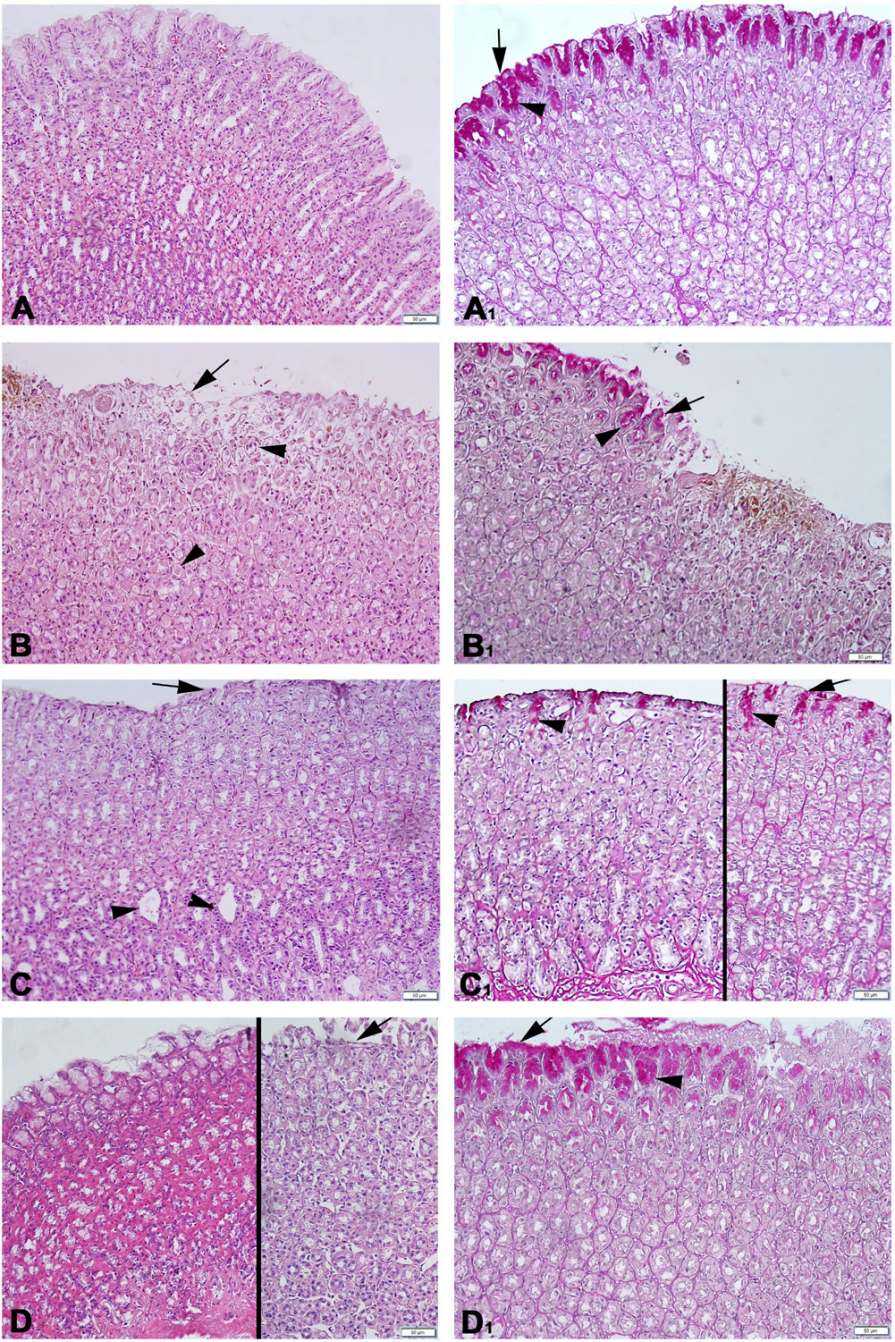
Representative micrographs illustrating the histological appearances of gastric samples from the indomethacin groups. Micrographs A, B, C, D depict H&E staining; A1, B1, C1, D1 depict PAS staining. Indo control group (A, A1) revealed a regular gastric surface epithelium and glandular cells with PAS-positive surface mucous cells (arrow) and neck mucous cells (arrowhead). In indo ulcer group (B, B1), there was severe degeneration in both surface cells (arrow) and glandular epithelial cells (arrow) with inflammatory cell infiltration (e). There was a significant decrease in PAS-positive surface mucous cells (arrow) and neck mucous cells (arrowhead). RTN-indo ulcer group (C, C1) revealed partly preserved surface epithelium (arrow) and glandular epithelium (arrowhead) with a moderate improvement in PAS-positive surface mucous (arrow) and neck mucous cells (arrowhead). MET-indo ulcer group (D, D1) was comparable to RTN-ethanol ulcer group in terms of morphology with more increase in PAS-positive surface mucous cells (arrow) and neck mucous cells (arrowhead). Scale: 50 µm. Original magnification: 10×

Gastric samples of the ethanol ulcer and indo ulcer groups revealed severely damaged mucous and glandular epithelium with diffuse hemorrhage and inflammatory cell infiltration (Figures 2 and 3). In these groups, Periodic Acid–Schiff (PAS)-positive areas were markedly decreased in damaged mucosal regions compared to control groups. As given in Table 1, the evaluation of lesions at microscopic level showed significantly increased scores in both ethanol ulcer and indo ulcer groups compared to their controls.

In MET-ethanol ulcer and MET-indo ulcer groups, the epithelial layer had mild damage of surface mucous cells, moderate damage of glandular epithelium, vascular congestion, and inflammatory cell infiltration. PAS-positive surface mucous cells and glandular epithelial cells were markedly increased in MET-ethanol ulcer group compared to ethanol ulcer group whereas it was slightly increased in MET-indo ulcer group compared to indo ulcer group (Figures 2 and 3). In the same parallel, the microscopic lesion score of metformin-treated ulcer groups was significantly lower compared to untreated ulcer groups (Table 1).

In ranitidine-treated ulcer groups, the microscopic evaluations were comparable to those of metformin-treated ulcer groups (Figures 2 and 3 and Table 1).

### Gastric Glutathione, Superoxide Dismutase, and Catalase Levels

No statistically significant difference was observed among ethanol groups in terms of gastric glutathione content; indo ulcer group presented reduced glutathione levels (0.35 ± 0.03 μmol/g) in comparison to control (0.65 ± 0.04 μmol/g; *P* < .01). This item was restored by both metformin (0.57 ± 0.03 μmol/g; *P* < .05) and ranitidine (0.57 ± 0.08 μmol/g; *P* < 0.05) treatments (Table 2).

Ethanol ulcer group revealed decreased gastric SOD activity (8.49 ± 0.71 U/mL) in comparison to control (12.57 ± 0.10 U/mL; *P* < .01). Pretreatment with metformin reversed this effect (11.73 ± 0.73 U/mL; *P* < .05). No significant difference was observed among indo control, indo ulcer, and MET-indo ulcer groups in terms of SOD activities whereas RTN-indo ulcer group showed a higher SOD activity compared to untreated group (Table 2).

Gastric catalase acitivities demonstrated no statistically significant change among groups in both ulcer models (Table 2).

### Chemiluminescence Levels

Both luminol- and lucigenin-enhanced chemiluminescence levels showed elevations in ethanol ulcer group and indo ulcer group compared to their controls. Metformin and ranitidine treatments were markedly effective to improve these values (Table 3).

### Evaluation of Apoptosis

In both ethanol- and indo-induced ulcer groups, the percentage of TUNEL-positive apoptotic cells was higher (10.67 ± 0.38% and 7.57 ± 0.42%, respectively) when compared to that of control groups (1.77 ± 0.14% and 1.47 ± 0.03%, respectively; *P* < .001). The level of apoptosis was restored in MET-ethanol ulcer (3.69 ± 0.19%; *P* < .001) and MET-indo ulcer groups (5.18 ± 0.54%; *P* < .001). Ranitidine treatment to rats in both groups reversed the apoptotic index near control levels (2.05 ± 0.17%; *P* < .001 for ethanol and 2.40 ± 0.24%; *P* < .001 for indo).

**Figure 4.**
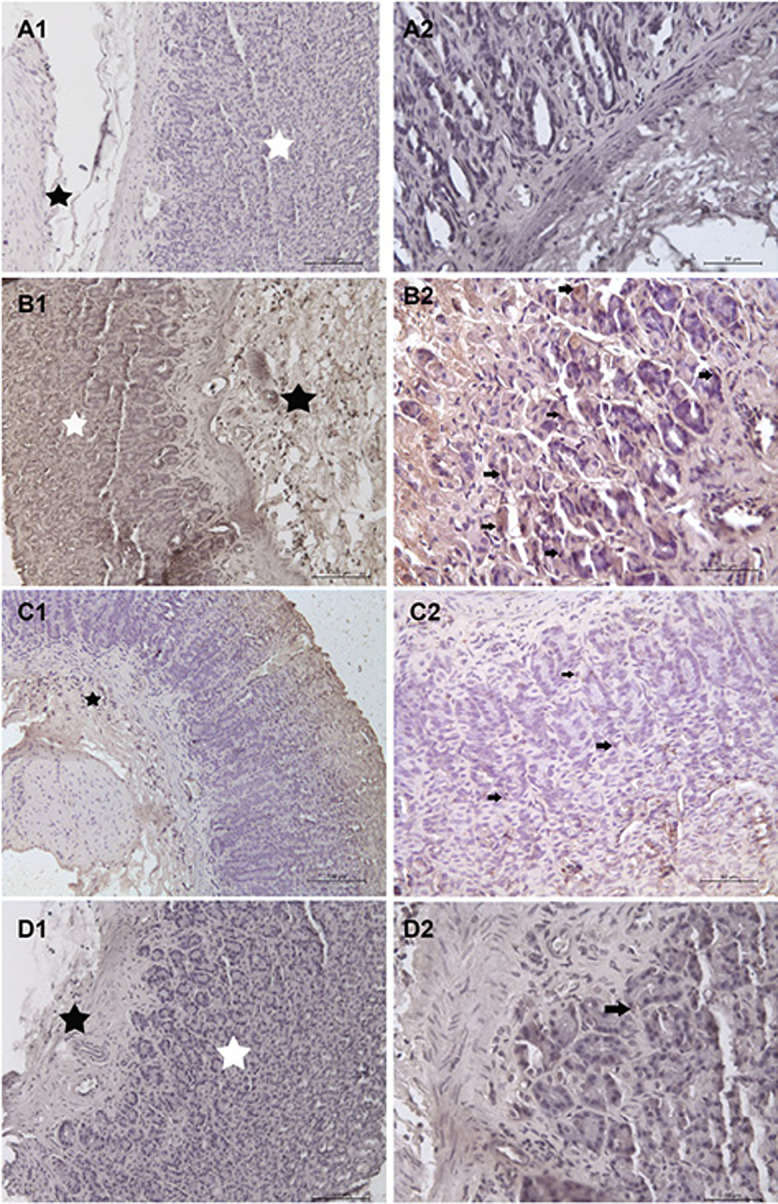
Immunohistochemical staining (TUNEL) images of the representative gastric samples from the experimental groups: ethanol control group (A1, A2), ethanol ulcer group (B1, B2), MET-ethanol ulcer group (C1, C2), and RTN-ethanol ulcer group (D1, D2). Black arrows demonstrate the apoptotic cells in tunica submucosa (black asteriks) and tunica mucosa (white asteriks). Scales and original magnification: 100 µm and 20× (A1, B1, C1, D1) and 50 µm and 40× (A2, B2, C2, D2).

**Figure 5.**
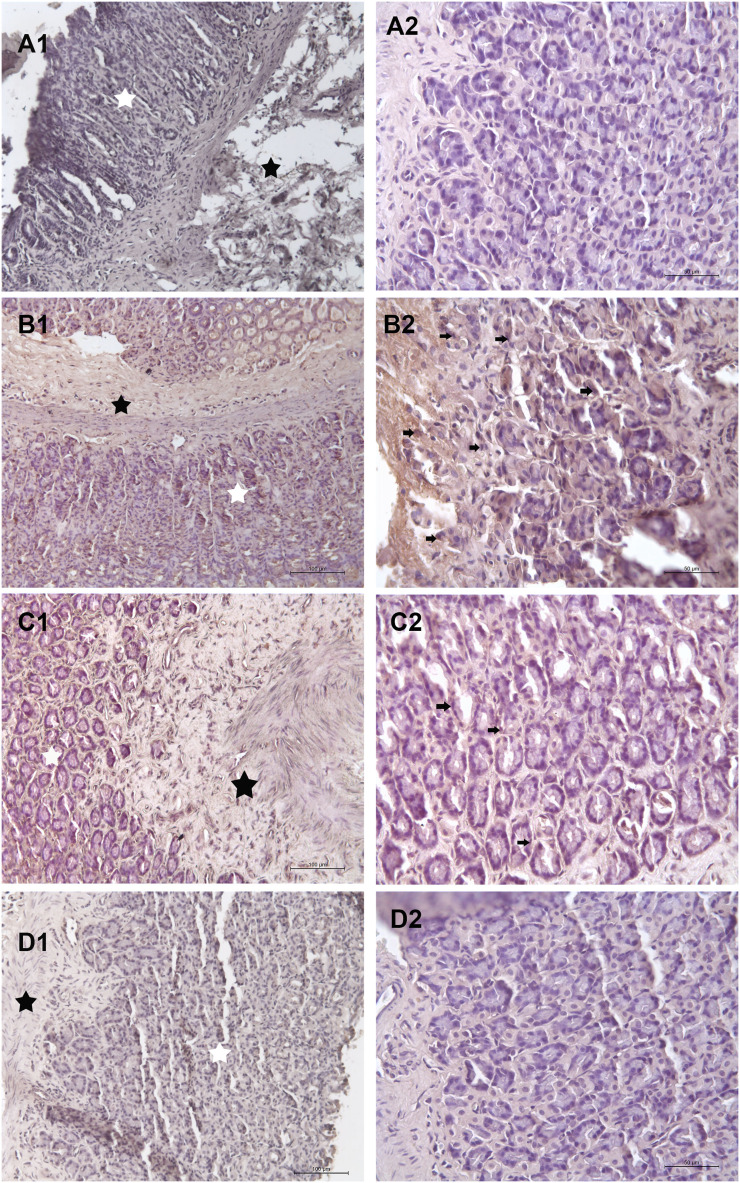
Immunohistochemical staining (TUNEL) images of the representative gastric samples from the experimental groups: indo control group (A1, A2), indo ulcer group (B1, B2), MET-indo ulcer group (C1, C2), and RTN-indo ulcer group (D1, D2). Black arrows demonstrate the apoptotic cells in tunica submucosa (black asterisks) and tunica mucosa (white asterisks). Scales and original magnification: 100 µm and 20× (A1, B1, C1, D1) and 50 µm and 40× (A2, B2, C2, D2).

As demonstrated in Figures 4 and 5, in both ethanol- and indo-induced ulcer groups, there was markedly increased TUNEL-positive cells in the gastric mucosa in comparison to their controls. Metformin pretreatment to both ulcer groups lowered TUNEL-positive cell population in comparison to untreated ones.

### Evaluation of the Gastric Blood Flow

**Figure 6.**
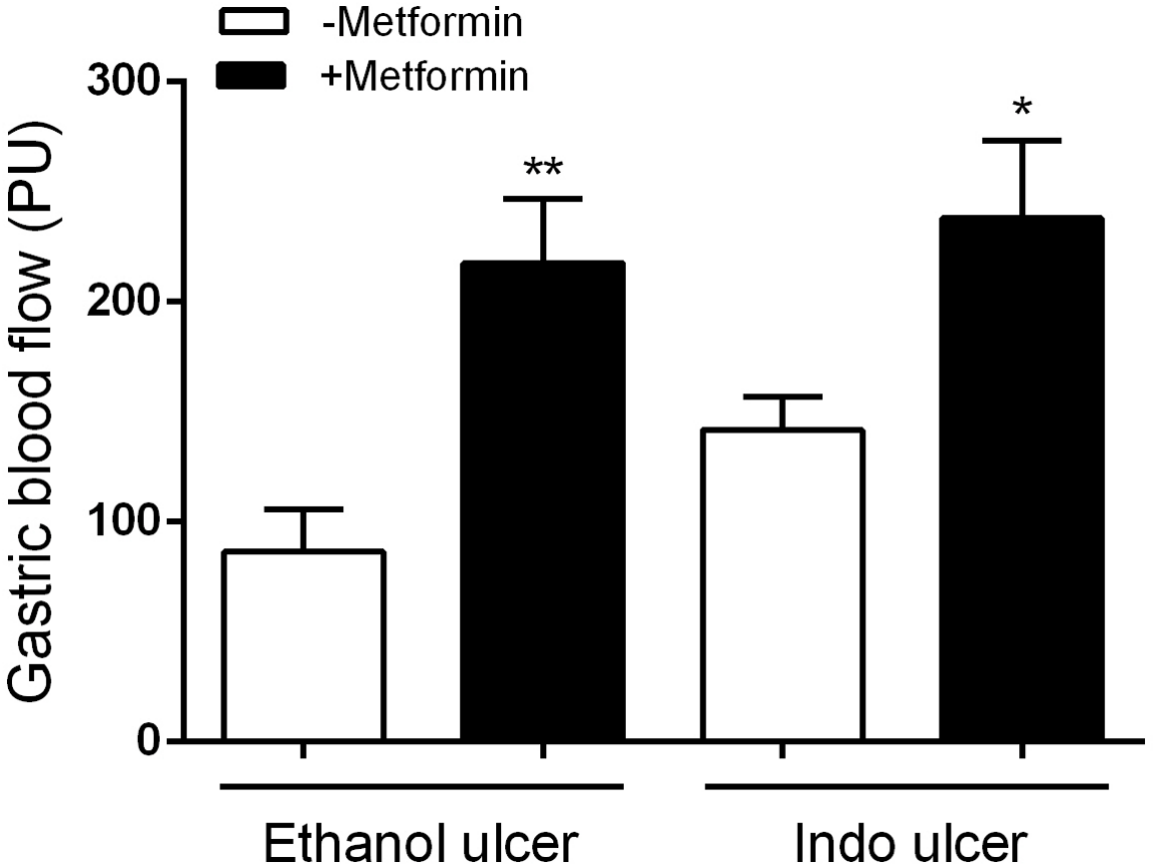
Gastric blood flow values of the experimental groups. Data are expressed as mean ± SEM. ^*^ *P* < .05 versus indo ulcer group; ^**^ *P* < .01 versus ethanol ulcer group.

Gastric blood flow analysis revealed elevated gastric blood flow levels in both MET-ethanol ulcer and MET-indo ulcer groups (217.70 ± 29.19 PU; *P* < .01 and 238.00 ± 35.49 PU; *P* < .05, respectively) compared to untreated ones (86.42 ± 19.32 PU and 141.90 ± 14.91 PU, respectively) (Figure 6).

### Serum IL-1β Levels

Serum IL-1β levels showed slight increases in both ethanol ulcer (0.37 ± 0.08 pg/mL) and indo ulcer groups (0.36 ± 0.05 pg/mL) in comparison to control groups (0.34 ± 0.05 pg/mL; *P* < .05 and 0.36 ± .05 pg/mL; *P* < .001, respectively). Metformin treatment had no significant effect on this parameter. Ranitidine decreased serum IL-1β level only in indo ulcer group (0.34 ± 0.07 pg/mL; *P* < .05).

## Discussion

This study investigated the impact of metformin, a widely used anti-diabetic drug, on two ulcer models in the rat independent of its effect on blood glucose levels. The findings of the study demonstrated severely damaged gastric mucosa together with impaired redox homeostasis and increased apoptosis in rats subjected to intragastric ethanol or indomethacin installations. These changes were accompanied by reduced endogenous antioxidants glutathione and SOD in indo ulcer and ethanol ulcer groups, respectively. Metformin treatment at a dose of 500 mg/kg orogastrically for 3 days prior to ethanol or indomethacin administrations was found to be effective to protect the stomach against these ulcerogenic agents.

Absolute ethanol given orally penetrates into the gastric mucosa and produces lesions that are characterized by extensive edema, hemorrhage, desquamation of the epithelial cells, and inflammatory cell infiltration as observed in humans and animal models.^20-22^ In accordance with other studies, in our study, the macroscopic and microscopic evaluations revealed a severely damaged gastric mucosa, elevated oxidant production, apoptosis, and decreased SOD activity along with high serum IL-1β levels in the ethanol ulcer group.

Prostaglandins stimulate gastric mucus and bicarbonate secretion, increase mucosal blood flow, and suppress inflammatory cell infiltration into the mucosa. Prostaglandins have an inhibitory action on the release of pro-inflammatory cytokines including tumor necrosis factor (TNF)-α, IL-1β and IL-8 which are known to promote epithelial cell apoptosis and activate adhesion molecules. There are several reports showing the development of gastrointestinal complications with the chronic use of NSAIDs in the clinical practice. In addition to blocking of prostaglandin synthesis by inhibition of cyclooxygenase enzymes, NSAIDs trigger the generation of ROS which impair membrane integrity via both peroxidation of membrane lipids and induction of epithelial cell apoptosis. In a recent study by Shahan et al.^23^ indomethacin upregulates the expression of caspase-3 mRNA levels approximately 3-fold as compared to normal group. In our study, increased oxidant production (assessed by chemiluminescence method) with concomitant reduction of endogenous antioxidant glutathione content and increased immunostained apoptotic cell population in the gastric samples of the indo ulcer group than control are in line with previous observations.

It is well known that prostaglandins provide gastroprotection via preserving microvascular integrity and maintenance of blood flow. In addition to the release of ROS, gastric blood flow stasis and microvascular disruption are also key factors for the mechanisms of ethanol-induced lesions, as stated by Szabo et al.^24^ gastric blood flow stasis may contribute to injury through increasing susceptibility of the mucosa to damage in both models. As described previously by Gana et al.^25^ aspirin induces focal ischemia in gastric mucosa and leads to gross morphological ulceration. This observation was also supported by Santos et al.^26^ showing a marked reduction in gastric blood flow and in gastric prostaglandin E2 (PGE2) levels in rats given indomethacin per oral at a dose of 20 mg/kg.

In various inflammatory settings, metformin was reported to have anti-inflammatory properties beyond its effect to control glycemia.^4,5,27^ It increases colonic SOD and catalase activities in acute colitis^28^ and is beneficial in chronic colitis via improving mucosal damage score at macroscopic and microscopic levels, decreasing the oxidative stress, and the inflammatory markers.^3^ In a study by Yanardag et al.^29^ it was demonstrated that metformin increased glutathione levels in the liver of diabetic rats. In a rat model of hepatic toxicity, treatment with metformin prevented liver dysfunction via activating antioxidant systems including SOD, catalase, and glutathione transferase enzymes.^30^ According to our findings, the decreases in gastric glutathione level in indo ulcer group and SOD activity in ethanol ulcer group were both reversed with metformin pretreatment. As stated by Malinska et al.^31^ the maintenance of redox balance is one of the key mechanisms of metformin as an anti-inflammatory agent. Metformin was shown to inhibit TNF-α-induced IL-1β and IL-8 expression and nuclear factor (NF)-κB signaling in human colon cancer cell line COLO 0205.^27^ The same study speculated that metformin might attenuate intestinal inflammation via adenosine monophosphate-activated protein kinase (AMPK) activation and subsequent NF-κB suppression. The NF-κB pathway, which initiates a strong inflammatory and immune response, stimulates the production of inducible nitric oxide synthase (iNOS), TNF-α, and IL-6. AbdelAziz et al.^32^ reported that metformin administration in indomethacin-induced gastric ulcer model reduced tissue NF-kB levels. In another study investigating the effect of metformin on gastric damage, it was suggested that the protective effect of metformin was attenuated by an AMPK inhibitor.^33^ Our study data and the findings of a study by Hosono et al.^34^ demonstrated that metformin (500 mg/kg per day) does not alter the blood glucose levels. Thus, its antioxidant and anti-inflammatory effects shown in these studies seem to be independent of its effect on glucose metabolism.

Elevated production of ROS mainly by inflammatory cells (e.g., neutrophils) migrated to the injured area and depletion of antioxidant enzymes due to the oxidant stress are common features of gastric ulcers.^35^ In our study, increased inflammatory cell infiltration was observed at microscopic examination and increased ROS generation was shown by chemiluminescence assay. In metformin-pretreated ulcer groups, both parameters were markedly restored.

Additionally, we observed a significantly elevated gastric blood flow in metformin-pretreated ulcer groups with respect to untreated groups. This suggests that metformin might facilitate tissue restoration indirectly by improving gastric stasis which may limit the contact of the mucosa with the irritant and/or buffer the back-diffusing acid. A previous study examining the protective effect of metformin against indomethacin-induced gastric ulcers in diabetic rats reported increased gastric mucosal nitric oxide (NO) levels compared with untreated diabetic rats with no change in gastric PGE2 levels.^36^ As NO is known to stimulate gastric mucus production and improve tissue perfusion via vasodilation, the beneficial effect of metformin may be partly attributed to its ability to stimulate NO synthesis. This issue deserves further investigation.

In conclusion, the current work demonstrated the gastroprotective effect of biguanide metformin against gastric ulcer development in ethanol- and indomethacin-challenged non-diabetic, normoglycemic rats. This effect seems to stem from its antioxidant and anti-apoptotic properties and partly from its ability to restore blood flow. The underlying mechanisms that will be further explored may highlight whether it might be useful as an anti-ulcer agent for diabetic and non-diabetic populations in the clinical setting.

## Figures and Tables

**Table 1.** Macroscopic Lesion Score, Lesion Length, and Microscopic Score of the Stomach in Experimental Groups

	Macroscopic Lesion Score	Lesion Length (mm)	Microscopic Lesion Score
**Ethanol groups**			
Ethanol control	0.01 ± 0.01	0.01 ± 0.01	0.01 ± 0.01
Ethanol ulcer	5.99 ± 0.01^***^	54.88 ± 8.11^***^	6.38 ± 1.19^***^
MET-ethanol ulcer	1.37 ± 0.90^+++^	2.38 ± 1.69^+++^	2.50 ± 0.82^++^
RTN-ethanol ulcer	3.29 ± 1.04^+^	22.71 ± 10.89^++^	2.00 ± 0.53^++^
**Indomethacin groups**			
Indo control	0.01 ± 0.01	0.01 ± 0.01	0.01 ± 0.01
Indo ulcer	5.99 ± 0.01^***^	69.31 ± 6.67^***^	5.25 ± 1.09^***^
MET-indo ulcer	4.50 ± 0.98^***^	38.63 ± 10.56^+^	2.43 ± 0.57^+^
RTN-indo ulcer	4.43 ± 0.65^***^	13.71 ± 4.71^+++^	2.29 ± 0.42^+^

Data are expressed as mean ± SEM.

Indo, indomethacin; MET, metformin; RTN, ranitidine.

^***^ *P* < .001 versus control groups; ^+^ *P* < .05, ^++^ *P* < .01, and ^+++^ *P* < .001 versus ulcer groups.

**Table 2.** Gastric Glutathione Content, SOD, and Catalase Enzyme Activities in Experimental Groups

	Glutathione (μmol/g)	SOD (U/mL)	Catalase (U/mL)
**Ethanol groups**			
Ethanol control	0.57 ± 0.07	12.57 ± 0.10	0.41 ± 0.09
Ethanol ulcer	0.59 ± 0.03^ns^	8.49 ± 0.71^**^	0.35 ± 0.09^ns^
MET-ethanol ulcer	0.57 ± 0.06^ns^	11.73 ± 0.73^+^	0.23 ± 0.08^ns^
RTN-ethanol ulcer	0.64 ± 0.07^ns^	10.68 ± 1.09^ns^	0.24 ± 0.06^ns^
**Indomethacin groups**			
Indo control	0.65 ± 0.04	10.64 ± 0.52	0.22 ± 0.05
Indo ulcer	0.35 ± 0.03^**^	9.73 ± 0.28^ns^	0.18 ± 0.03^ns^
MET-indo ulcer	0.57 ± 0.03^+^	10.84 ± 0.63^ns^	0.35 ± 0.09^ns^
RTN-indo ulcer	0.57 ± 0.08^+^	13.56 ± 0.43^+++^	0.29 ± 0.07^ns^

Data are expressed as mean ± SEM.

Indo, indomethacin; MET, metformin; RTN, ranitidine; SOD, superoxide dismutase.

^**^P < .01 versus control groups; ^+^*P* < .05 and ^+++^*P* < .001 versus ulcer groups; ns, non-significant.

**Table 3.** Luminol- and Lucigenin-Enhanced CL Levels of the Gastric Samples in Experimental Groups

	Luminol-Enhanced CL (rlu/mg)	Lucigenin-Enhanced CL (rlu/mg)
**Ethanol groups**		
Ethanol control	29.05 ± 3.01	30.01 ± 3.40
Ethanol ulcer	59.71 ± 5.87^***^	52.56 ± 4.12^**^
MET-ethanol ulcer	27.15 ± 1.32^+++^	37.33 ± 4.64^+^
RTN-ethanol ulcer	28.97 ± 3.01^+++^	31.76 ± 3.02^++^
**Indomethacin groups**		
Indo control	34.65 ± 4.18	24.23 ± 3.11
Indo ulcer	64.69 ± 7.06^***^	59.01 ± 7.98^***^
MET-indo ulcer	37.00 ± 4.05^++^	38.54 ± 4.39^+^
RTN-indo ulcer	28.67 ± 1.97^+++^	25.03 ± 1.63^+++^

Data are expressed as mean ± SEM.

CL, chemiluminescence; Indo, indomethacin; MET, metformin; RTN, ranitidine.

^**^P < .01 and ^***^P < .001 versus control groups; ^+^*P* < .05, ^++^*P* < .01, and ^+++^*P* < .001 versus ulcer groups.
